# Synergistic Effect between Amoxicillin and Zinc Oxide Nanoparticles Reduced by Oak Gall Extract against *Helicobacter pylori*

**DOI:** 10.3390/molecules27144559

**Published:** 2022-07-17

**Authors:** Hany G. Attia, Hassan A. Albarqi, Ismail G. Said, Omaish Alqahtani, Mohamed A. EI Raey

**Affiliations:** 1 Department of Pharmacognosy, College of Pharmacy, Najran University, Najran 1988, Saudi Arabia; osalqahtani@nu.edu.sa; 2 Department of Pharmaceutics, College of Pharmacy, Najran University, Najran 1988, Saudi Arabia; haalbarqi@nu.edu.sa; 3 Department of Chemistry of Natural and Microbial Products, National Research Centre, Dokki, Cairo 12311, Egypt; ismailsaid66@yahoo.com; 4 Department of Phytochemistry and Plant Systematics, National Research Centre, Dokki, Cairo 12311, Egypt

**Keywords:** zinc oxide nanoparticles, *Oak galls*, LC-MS/MS, *Helicobacter pylori*, checkerboard assay

## Abstract

*Helicobacter pylori* (*H. pylori*) is a global health threat, and the World Health Organization has included *H. pylori* among 12 bacterial species that require high priority future strategies for the development of new antibiotics due mainly to its high rates of resistance. Metallic nanoparticles are known for their antimicrobial properties. The FDA (Food and Drug Administration) has approved zinc oxide nanoparticles (ZnONPs) as biocompatible antimicrobials. Green synthesis of ZnONPs was performed based on *Oak* galls extract (OGE) and was characterized by UV, IR, DLS, TEM, and SEM measurements. In addition, LC-MS/MS was used for the identification of OGE constituents. A checkerboard assay was used to evaluate the activity of synthesized Qi-ZnONPs and OGE against *H. pylori*, and their synergistic effects with amoxicillin were evaluated. LC-MS/MS analyses identified 20 compounds as major gallic acid conjugates. The ZnONPs had average particle sizes of 5.5 nm (DLS) and 7.99 nm (TEM). Both OGE and Qi-ZnONPs exhibited moderate activity against *H. pylori*. Amoxicillin and Qi-ZnONPs combinations (1:2 and 1:4 amoxicillin:/Qi-ZnONPs) significantly decreased the MIC_90_ by two-fold and four-fold, respectively, and FIC values for the combinations were more significant than with OGE alone. OGE is rich in phenolics. The synergism between Qi-ZnONPs and amoxicillin can provide an alternative safe agent of low cost to combat *H. Pylori* infections.

## 1. Introduction

*Helicobacter pylori* is a spiral Gram-negative bacterium that colonizes the human gastric mucosa and has infected over half of the world’s population. Chronic stomach inflammation caused by *H. pylori* can develop into peptic ulcers, MALT-lymphoma, or gastric adenocarcinoma, the latter of which is the most significant cause of cancer-related deaths worldwide. According to a recent study, *H. pylori* infected people had a six-fold higher risk of stomach cancer than *H. pylori* negative people [1]. The rapid rise of *H. pylori* strains that are resistant to traditional antibiotic therapy, which has been reported worldwide, is concerning and highlights the urgent need to develop new resistance-prevention strategies [2]. Furthermore, the World Health Organization has designated *H. pylori* as one of 12 bacterial species that require high priority future plans to develop new antibiotics, owing to high resistance rates [3]. Amoxicillin is a common antibiotic that is used to treat a variety of bacterial illnesses. Amoxicillin and clarithromycin are also used to treat stomach ulcers caused by *H. pylori* infection [4].

Mabeku et al. 2019 used 140 *H. pylori* isolates that were collected from stomach biopsies of dyspeptic patients in two Cameroonian reference hospitals to evaluat their antimicrobial susceptibility to amoxicillin. Amoxicillin resistance was observed in 97% of *H. pylori* strains [5]. It was found that combining ZnONPs with antibiotics such as Metronidazole had a synergistic impact against *H. pylori* due to the nanoparticles’ ability to permeate membranes and cause damage, allowing the medicines to reach their intracellular targets more effectively [1]. The antimicrobial properties of nanoparticles (NPs) have long been known [6]. Metal and metal oxide nanoparticles are particularly interesting among inorganic nanomaterials because they display potent antibacterial activity even at extremely low concentrations. Because of their biocompatibility and resilience, ZnONPs are becoming more popular due to their toxicological safety. This substance was recently authorized as GRAS (generally recognized as safe) by the US Food and Drug Administration. ZnONPs have been shown to have antibacterial activity against a wide range of Gram-positive and Gram-negative bacteria [1]. Furthermore, nanoparticles made from plant extracts are of interest because they are less harmful, cost-effective, and environmentally friendly [7]. *Quercus infectoria* is a tree that is a member of the Fagaceae family. It grows in Egypt and can be found in North Africa, Turkey, Asia (Syria and Iran), and South Europe (Cyprus and Greece). Gallotannis, ellagic acid, starch, and sugar are the primary components of *Quercus infectoria* galls [8]. Galls have been used in folk medicine to restore postpartum uterine elasticity, motivate vaginal discharges, as a component of toothpaste for treating oral cavity diseases, and to treat diarrhea, dysentery, internal hemorrhages, painful gonorrhea, impetigo, tonsillitis, and menorrhagia. Pharmacologically, OGEs have exhibited anti-diabetic, antibacterial, antiviral, antifungal, larvicidal, anti-inflammatory, anti-amoebic, and wound healing properties [8,9].

Our goals in this study are to identify the chemical constituents of OGE using LC-MS/MS, synthesize ZnO nanoparticles based on OGE, evaluate their activity against *H. pylori,* and study their synergistic activities using checkerboard assays with inexpensive antibiotics such as amoxicillin.

## 2. Results and Discussion

### 2.1. High-Performance Liquid Chromatography- Mass Spectrometry Analysis (LC-MS/MS)

**Figure 1:**
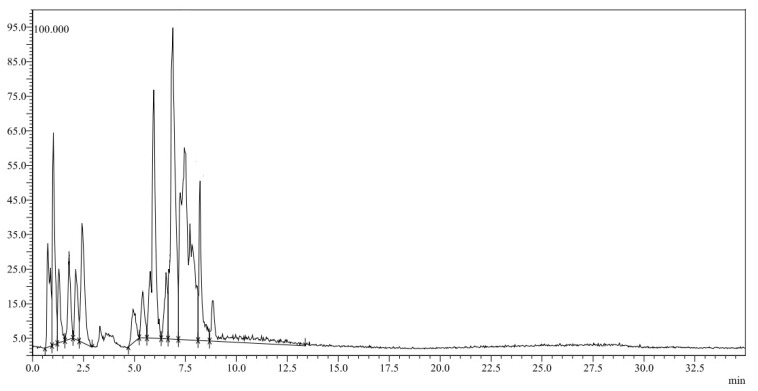
LC/MS chromatogram of a *Q. infectoria galls* ethanol extract.

LC-MS/MS was used to characterize the OGE as shown in Figure 1. which will be used as reducing and capping agents for the preparation of green nanoparticles.

Phytochemical investigation using LC-MS/MS of OGE led to the identification of 20 phenolic metabolites which are listed in Table 1. These metabolites were identified by comparing their fragmentation pattern with those reported in the literature. Most of these metabolites are hydrolysable tannins, especially gallotannins: galloyl glucoses; gallic acid and methyl gallate. They also include gallic acid dimers (digallic acids and their methyl esters), gallic acid trimers (trigallic acid and its methyl ester), and phenolic acids, such as di-hydroxybenzoic and syringic acids. Galloyl glucoses (Mono, di, tri, tetra and penta), ellagic acid, syringic acid, and methyl gallate were identified previously in *Q. infectoria* galls. 

**Figure 2:**
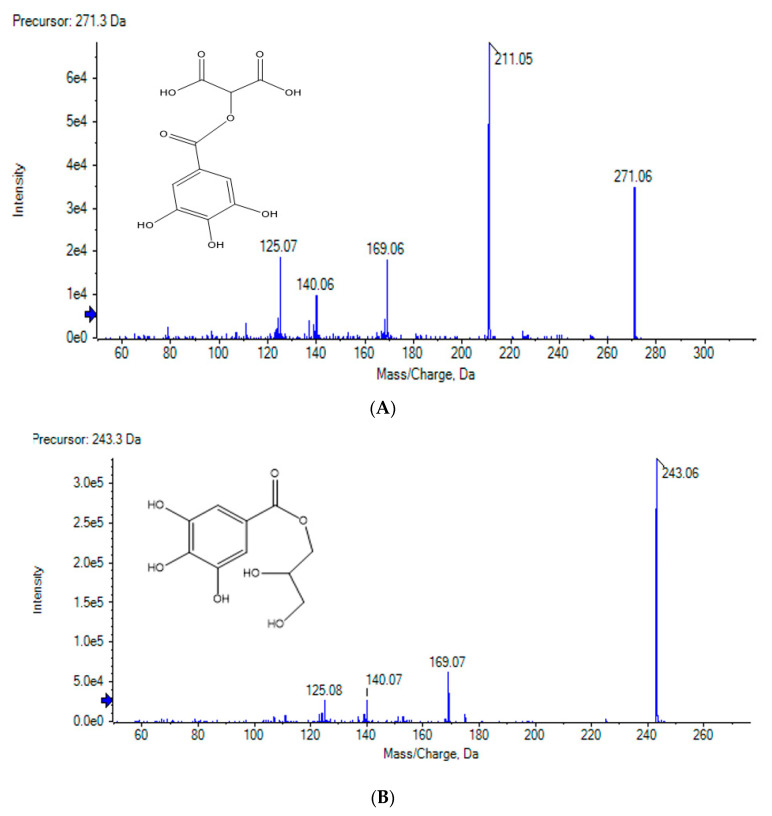
(**A**) LC-MS/MS of Compound 2 (2-*O*-galloyl hydroxy malonic acid); (**B**) LC-MS/MS of Compound 6 (Gallic acid glyceride).

Compound 2 has a molecular ion of [M-H]- at m/z 271 with a fragment at m/z 169 [M-H-102]- suggesting that 2 is 2-O-galloyl hydroxymalonic acid. Compound 6 has a molecular ion of [M-H]- at m/z 243 with a fragment at m/z 169 [M-H-74]- suggesting that 6 is galloyl glyceride. (Figure 2)

### 2.2. Preparation of Nanoparticles

The reduction of zinc sulfate with an ethanolic extract of *Q. infectoria* galls on heating, followed by the addition of a few drops of ammonia, led to the precipitation of zinc oxide nanoparticles [20].

### 2.3. Ultraviolet Analysis (UV)

UV analysis of the synthesized Qi-ZnONPs showed peaks at 278 and 358 nm (Figure S1). These data are consistent with those reported previously for the preparation of Qi-ZnONPs [21].

### 2.4. FT-IR of Q.infectoria Galls and Synthesized Qi-ZnONPs

**Figure 3:**
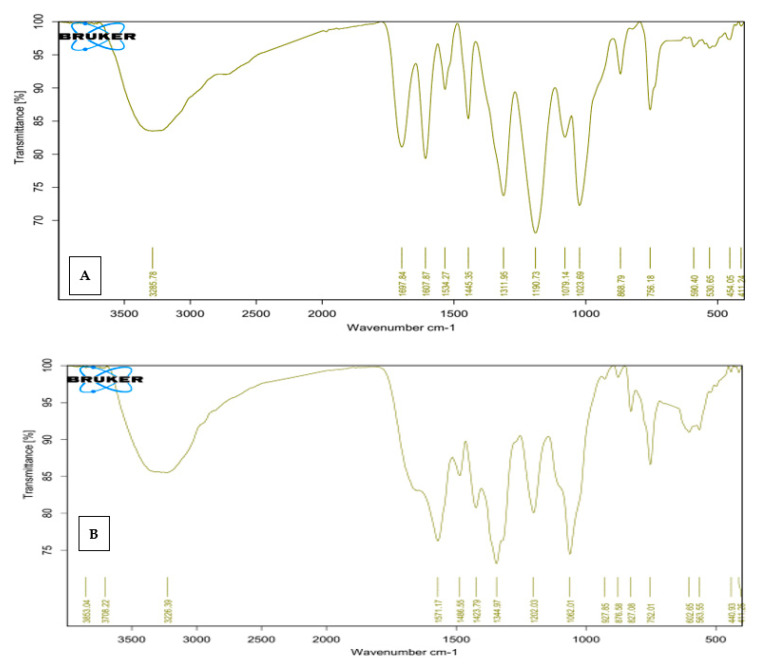
FT-IR spectra of *Q.infectoria* galls extract (**A**) and synthesized Qi-ZnO-NPs (**B**).

FT-IR spectra (Figure 3A,B) were obtained from 400 to 4000 cm^−1^ to identify the different functional groups present in *Q. infectoria* and Qi-ZnONPs. As shown in (Figure 3B), there is a difference (relative to Figure 3A) in *Q. infectoria* galls; this is due to the formation of ZnO nanoparticles.

FT-IR spectra (Figure 3B) confirm the synthesis of Qi-ZnONPs by the bending vibrations of Zn-O at 441 and 593 cm^−1^. The band in the region 3226 cm^−1^ can be assigned to the presence of water molecules on the surface of Qi-ZnONPs (O–H stretching vibration) [22].

### 2.5. Transmission Electron Microscope (TEM) Analysis

**Figure 4:**
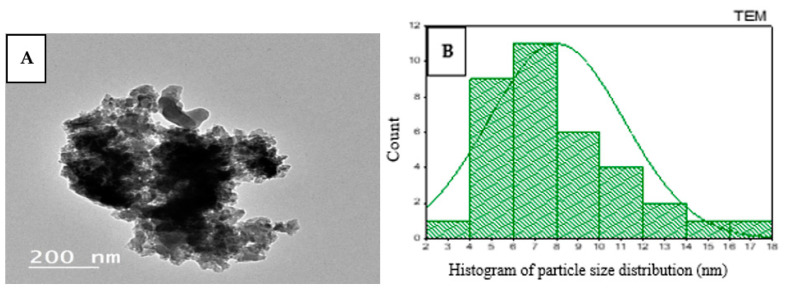
TEM image of Qi-ZnO-NPs (**A**) and a particle size distribution histogram (**B**).

TEM analysis showed the presence of amorphous particles (Figure 4A). The particle size distribution is shown in Figure 4B, and its average size was calculated to be 7.99 ± 3.14 nm. 

### 2.6. Scanning Electron Microscope Analysis (SEM)

**Figure 5:**
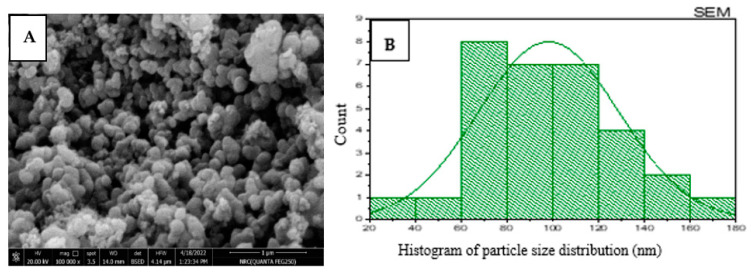
SEM image of Qi-ZnO-NPs (**A**) and particles size distribution histogram (**B**).

SEM analysis was applied to study the morphological structure of green prepared nanoparticles Figure 5A,B. The particles are clearly spherical structures. The Qi-ZnONPs nanoparticles exhibit particle sizes of 20–180 nm. The homogenous distribution of particles provides a good description of the nature of the particle’s sizes. Previous literature results indicate that nanoparticles that used natural products as reducing and capping agents led to particle agglomeration. Therefore, the particles appear to have larger particles sizes [23].

### 2.7. Dynamic Light Scattering Measurements (DLS)

Particle size measurements of Qi-ZnONPs (Figure S2A) were performed in 70% aq. ethanol and demonstrated that Qi-ZnONPs produced two peaks, namely one at 8.062 nm with an intensity of 97.4%, and another peak at 3871 nm with an intensity of 2.4%. The average particle size was 5.541 nm with a PDI of 0.322. Furthermore, the zeta potential diagram showed that these particles were stable with a value of –24.7 mV (Figure S2B).

### 2.8. Evaluation of Anti-Helicobacter Pylori Activity

The antibacterial activity of various transition metal oxide nanostructures arises from an electrostatic induction between the NPs and the bacterial cell membrane [24]. The NPs remained on the surface of the bacteria for extended time and did not penetrate the membrane. The NPs alter the viscosity of the membrane and impair specific ionic pumps, and eventually affect transport exchanges between the bacterial cell and the solution to disturb bacterial growth [25]. The synergetic effects of Qi-ZnONPs resulted from oxidative stress. Zn^2+^ ions can be absorbed by bacterial cells and can inhibit the action of respiratory enzymes in the cell membrane by interacting with them. Oxidative stress is also key factor in promoting antibacterial activity. Oxidative stress can be induced by the generation of reactive oxygen species in vivo, which can cause oxidative stress and lead to irreversible damage to the bacterial cell [26]. The increased particle surface area, reduced band gap energy, and improved adsorption ability of the particle surfaces can attack nucleic acids, proteins, polysaccharides, lipids, and other biological molecules causing a loss of their function and eventually killing and decomposing the bacteria [27]. In addition, they can induce the hydrolysis of DNA into fragments. 

An agar diffusion method according to the clinical and laboratory standard institute (CLSI) was used to study the effect of OGE and Qi-ZnONPs on *H. pylori* ATCC-43526, compared to amoxicillin and clarithromycin as standard antibiotics (Table 2). 

OGE and Qi-ZnONPs exhibited anti-*H. pylori* (ATCC- 43526) activity in vitro as indicated by the inhibition zone diameter were 16 and 21 mm, respectively, compared to 28 and 31 mm obtained with amoxicillin and clarithromycin, respectively (Table 2). These results indicated that both OGE and Qi-ZnONPs have moderately sensitive activity against *H. pylori* compared to the antibiotics used in treatments.

The average MIC_90_s values for the OGE and the Qi-ZnONPs against *H. pylori* (ATCC-43526) were 37.5 and 18.75 µg/mL, respectively. In comparison, The MIC_90s_ for amoxicillin and clarithromycin (positive controls) were 0.586 and 0.293 µg/mL, respectively (Table 3). The inhibition of *H. pylori* growth in the MIC broth was calculated using the optical density (OD) values after 72 h of incubation for quality, negative, and blank controls, that contained no *H. pylori* or antimicrobials, only *H. pylori* (no antimicrobials) and only antimicrobials (no *H. pylori*), respectively, and clarithromycin as a positive control in parallel. The results demonstrated that the Qi-ZnONPs produced greater inhibition (98.4%) than the standard antibiotics, amoxicillin (93.2%) and clarithromycin (90.7%). These results demonstrated that the Qi-ZnONPs display a significant inhibitory effect on *H. pylori* growth and encourage further investigations of the synergistic effects of the Qi-ZnONPs in different combinations with amoxicillin as an economical antibiotic.

The synergistic effects of amoxicillin combined with both OGE and Qi-ZnONPs, individually against *H. pylori* were investigated using the checkerboard method.

As shown in Table 4, different combinations of amoxicillin and OGE (1:1 and 1:2) had no effect on the MIC values, but combinations of 1:4 and 1:8 decreased the MIC_90_ 2-fold, from 37.50 to 18.75 µg/mL, leading to a decrease in the FIC values of two-fold from 2.25 to 1.13. However, these combinations are not active against *H. pylori*.

Alternatively, the MIC values obtained with combinations of amoxicillin with Qi-ZnONPs were significantly different. The MIC_90_ decreased two-fold from 18.75 to 9.38 µg/mL, and four-fold from 18.75 to 4.69 µg/mL for amoxicillin: Qi-ZnONPs (1:1, 1:4), respectively. In addition, the FIC values became more significant for combinations of Qi-ZnONPs than for OGE, as they decreased the FIC values 8-fold from 2.25 (Indifference) to 0.282 (synergy) for amoxicillin: extract (1:1) and amoxicillin: Qi-ZnONPs (1:4), respectively, indicating that the combination between amoxicillin and the Qi-ZnONPs and the extract had a synergistic effect. The FIC value was 0.282 for the reference strain ATCC-43526, which is considered as synergy. For amoxicillin combined with OGE, the FIC value was 2.25 for the reference strain ATCC-43526, which is considered as indifferent. These results strongly confirm the potential for using Qi-ZnONPs in combination with the inexpensive antibiotic amoxicillin to provide alternative and low-cost strategies to combat *H. pylori* infections.

In conclusion, the Qi-ZnONPs have a strong and dose dependent activity against *H. pylori* ATCC-43526. The synergism between the nano form and a low-cost antibiotic can provide an additional advantage for evaluating *Q. infectoria* as a candidate for the treatment of *H. pylori* infections.

## 3. Materials and Methods

### 3.1. Plant Materials and Extraction Methods

The plant materials were purchased from a local market and authenticated by Dr. Mohamed El Gebally, former researcher of plant taxonomy at the National Research Centre. Powdered *Q. infectoria* galls (100 g) were extracted with 1 L of ethanol using an ultrasonic-assisted extraction method. The extract was filtered and evaporated under vacuum by rotavapor to yield 18.5 g of the extract.

### 3.2. High-Performance Liquid Chromatography-Mass Spectrometry Analysis (LC-MS/MS)

HPLC-MS/MS spectra were obtained using a Shimadzu 8045 LC system coupled with a mass spectrometer. LC was conducted using a UPLC—RP-C18 column (2.1×50 mm, particle size 1.7 µM). A gradient elution was applied that consisted of 0.1 % water and acetonitrile (ACN), each containing 0.1% formic acid, and the ACN was increased from 10% to 80% within 26 min and then to 10% within the next 5 min at a flow rate of 1 mL/min and a 1:1 split before the ESI source.

### 3.3. Green Synthesis of Zinc Oxide Nanoparticles

Nanoparticles of Qi-ZnONPs were biosynthesized using a previously described method [28], in which one gram of the *Q. infectoria galls* alcoholic extract was mixed with five grams of a zinc sulfate solution (in 500 mL of bi-distilled H_2_O) then heated for 20 min at 80 °C with stirring, followed by the addition of a few drops of an ammonia solution until a grey-white precipitate was formed. The reaction mixture was held for 30 min to complete the reaction. The precipitate that formed was collected by centrifugation at 4000 rpm and then washed two times with distilled water then once with ethanol to produce a grey-white powder of Qi-ZnONPs.

### 3.4. Characterization of Qi-ZnONPs

#### 3.4.1. Ultraviolet-Visible Spectral Analysis (UV)

A Shimadzu UV-1601 ultraviolet spectrophotometer (Shimadzu-Kyoto, Japan) was used to analyze preparations of Qi-ZnONPs. The UV spectra were obtained from 200 to 400 nm.

#### 3.4.2. Fourier Transform Infrared (FT-IR) Analysis

The Qi-ZnONPs functional groups were identified using an FT-IR 6100 spectrometer (Bruker-Billerica, Billerica, MA, USA) in the range of 4000–400 cm^−1^.

#### 3.4.3. DLS Measurements

Biosynthesized *Q. infectoria* zinc oxide nanoparticles’ particle size, stability, and charges were obtained using a zetasizer nano zs (Malvern, Worcestershire, UK).

#### 3.4.4. TEM

The morphology and particle sizes of Qi-ZnONPs were examined by TEM (JEOL-JEM-1011, Tokyo, Japan). A few drops of a suspension containing Qi-ZnONPs were placed on a carbon-coated copper grid, and the solvent was allowed to drain slowly before the TEM image was recorded.

#### 3.4.5. SEM

The topography of Qi-ZnONPs was investigated by SEM (Quanta FEG-250, FEL, Hillsboro, OR, USA).

### 3.5. Biological Studies

#### 3.5.1. Antibacterial Activity

The *H. pylori* strain used in this study was ATCC-43526 and was handled according to the supplier’s product information sheet. A vial of the lyophilized strain was thawed, and the entire content was inoculated aseptically into 5 mL of ATCC^®^ medium broth containing 6% defibrinated sheep blood and mixed well. It was then incubated at 37 °C under microaerophilic conditions using an anaerobic jar. Within 3–5 days of incubation, good growth was obtained. The *H. pylori* solution was added to cryopreservation media containing brain heart infusion broth with 25% glycerol and then stored at −80 °C.

#### 3.5.2. Inoculum Preparation

MIC susceptibility testing requires the use of standardized inocula. The 0.5 McFarland standards are recommended for use in the preparation of inocula for performing the MIC susceptibility test.

Stock cultures of *H. Pylori* were maintained at 4 °C on slopes of trypticase soy agar with 6% defibrinated sheep blood. Active cultures for experiments were prepared by transferring a portion of the cells from the stock cultures to test tubes containing 0.85% normal saline to produce a suspension of 0.5 McFarland turbidity standards (approximately 1.5 × 10^8^ CFU/mL). The turbidity of inoculated cultures was measured using a spectrophotometer at an OD of 540 nm (Agilent carry 100 UV/VIS Spectrometer, Range: 200–800 nm, Agilent, Santa Clara, California, USA). The solution was then diluted 50-fold to produce a turbid suspension containing approximately 3.0 × 10^6^ CFU/mL of *H. pylori*.

#### 3.5.3. Determination of Antibacterial Activities of the Suspended Samples

An agar well diffusion technique [29] was used to determine the in-vitro antibacterial activity of the powder samples as previously described [29]. A 0.1 mL aliquot of a 72 h broth culture of *H. pylori* ATCC-43526 that had been adjusted to a turbidity equivalent of 0.5 McFarland standards [30,31] was added to sterile Petri dishes previously labeled with *H. pylori* ATCC-43526. Molten sterile Muller-Hinton agar was poured aseptically onto the plates and gently rotated to permit the bacteria to be homogeneously distributed in the medium. The agar plates were allowed to solidify, after which equal amounts of 15 powder samples were placed as disks on the agar surfaces and the plates were allowed to diffuse for 2 h at 4 °C.

The experiment was conducted in duplicate. All plates were incubated at 37 °C for 72 h. Clearance zones around the wells were noted and measured in millimeters. Microorganisms that produced zone diameters ≥28 mm were classified as strongly sensitive, zone diameters of <28 to 16 mm as moderately sensitive, zone diameters of <16 to 12 mm as weakly sensitive, and zone diameters <12 mm as resistant [32].

#### 3.5.4. Susceptibility Testing

The minimum inhibitory concentration (MIC) was used to evaluate the inhibitory abilities of the antimicrobials, phytochemicals, nano biomaterials, etc., as antibiotics against *H. pylori*. The MIC of the assayed phytochemical extracts and their nano forms were determined using a macro dilution method. Sterile 10 mL test tubes containing 2 mL of sterile trypticase soy broth (Merck, Rahway, NJ, USA) were used to dilute the concentrated antibiotics ampicillin and clarithromycin (St. Louis, MO, Sigma, USA), phytochemical extracts, and nano form solutions with two-fold dilutions to obtain solutions with concentrations of 100, 50, 25, 12.5, and 6.25 mg/mL. Ten microliters of the *H. pylori* 0.5 McFarland inoculum were added to each phytochemical solution and then incubated at 37 °C. The OD values were measured using a spectrophotometer at an absorbance of 540 nm after 72 h. These experiments were performed with quality, negative and blank controls, which contained no *H. pylori* or antimicrobials, only *H. pylori* (no antimicrobials), or only antimicrobials (no *H. pylori*), respectively, and clarithromycin was used as a positive control in parallel. To avoid potential optical interference by the light scattering properties of samples particles during optical measurements of the growing cultures, the same liquid medium without microorganisms, but containing the same concentration of samples particles were cultured under the same conditions as the blank controls.

The MIC value for clarithromycin was calculated for comparison with those of the antimicrobials. All experiments followed the guidelines of the Clinical and Laboratory Standards Institute. The MIC_90_ value is the minimum concentration of phytochemicals that inhibit 90% of *H. pylori*. The percent inhibition was calculated using the following equation:

(1) $$Inhibition,\;\%=\frac{\mathrm{OD}\;\mathrm{value}\;\mathrm{of}\;\mathrm{the}\;\mathrm{sample}\;-\;\mathrm{OD}\;\mathrm{value}\;\mathrm{of}\;\mathrm{blank}\;\mathrm{control}}{\mathrm{OD}\;\mathrm{value}\;\mathrm{of}\;\mathrm{the}\;\mathrm{negative}\;\mathrm{control}\;-\;\mathrm{OD}\;\mathrm{value}\;\mathrm{of}\;\mathrm{quality}\;\mathrm{control}}\times 100$$

#### 3.5.5. Synergistic Testing

The synergistic effects of combinations between the extracts, the extracts nanoforms, and the antibiotics were evaluated using a checkerboard method as previously described [33].

Synergistic testing was used to determine if the additive effect of two antimicrobials or antibiotics was superior to that obtained with the individual antimicrobials. An additive effect may help to reduce the dose of each antimicrobial and may reduce adverse therapeutic effects. Here, the MIC values of the phytochemicals and antibiotics obtained from the susceptibility testing were used to calculate the concentrations required for synergistic testing. Various combinations of two antimicrobials (phytochemical extract and its nano form) with amoxicillin at different concentrations were evaluated. Their concentrations were 1:1 MIC, 1:2 MIC, 1:4 MIC, 1:8 MIC, antimicrobial alone, and amoxicillin alone. Checkerboard methods were used to calculate the fractional inhibitory concentration (FIC) values for the antimicrobials and amoxicillin against *H. pylori*. FIC values are measurements of synergistic, additive, or antagonistic effects.

An FIC value of less than 0.5 indicates a synergistic action, between 0.5 and 1.0 indicates additive action, 1.1–4.0 indicates indifference, and greater than 4.0 indicates antagonism.

All of the tests that were used to calculate FIC and MIC values were performed in the same manner. The FIC values were calculated using the following equation:

(2) $$\;\mathrm{FIC}\;\mathrm{value}\;=\frac{\mathrm{MIC}\;\mathrm{of}\;\mathrm{compound}\;A\;\mathrm{in}\;\mathrm{combination}\;}{\mathrm{MIC}\;\mathrm{of}\;\mathrm{compound}\;A\;\mathrm{alone}}+\frac{\mathrm{MIC}\;\mathrm{of}\;\mathrm{compound}\;B\;\mathrm{in}\;\mathrm{combination}\;}{\mathrm{MIC}\;\mathrm{of}\;\mathrm{compound}\;B\;\mathrm{alone}}$$

## 4. Conclusions

*Quercus infectoria* gall extracts are rich in phenolic metabolites, especially gallotannins. These phenolic metabolites are good reducing, capping, and stabilizing agents for the preparation of zinc oxide nanoparticles. Although both OGE and Qi-ZnONPs exhibited moderate anti- H. pylori activity, the Qi-ZnONPs exhibited greater inhibition (98.4%) than the standard antibiotics amoxicillin (93.2%) and clarithromycin (90.7%). The MIC_90_ for amoxicillin combinations with OGE was not significantly different, while the MIC_90_ values for amoxicillin combinations with Qi-ZnONPs were significantly different. The MIC_90_ decreased two-fold from 18.75 to 9.38 µg/mL, and two-fold from 18.75 to 4.69 µg/mL for amoxicillin: Qi-ZnONPs (1:1 and 1:4, respectively). The amoxicillin: Qi-ZnONPs (1:4) combination is a promising candidate for further studies for use as an economical, effective, and safe anti-*H. pylori.* drug

## Figures and Tables

**Table 1:** LC-MS/MS analysis of phenolic metabolites from *Q. infectoria galls* ethanol extract.

No	Rt	[M-H]^-^	MS/MS	Proposed Structures	Reference
1	0.75	191	173, 147	Quinic acid	[10]
2	0.79	271	169, 125	2-*O*-galloyl hydroxymalonic acid	-
3	0.92	331	169	Monogalloyl glucose	[11]
4	1.02	169	125	Gallic acid	[12]
5	1.04	153	109	Dihydroxy benzoic acid	[13]
6	1.21	243	169, 125	Galloyl glyceride	-
7	1.29	483	331, 169	Digalloyl glucose I	[14]
8	1.79	483	331, 169	Digalloyl glucose II	[14]
9	2.13	321	277, 233	*m*-digallic acid	[15]
10	2.44	321	277, 233	*P*-digallic acid	[15]
11	2.46	183	168, 125	Methyl gallate	[11]
12	4.95	635	483, 331, 313, 169, 125	Trigalloyl glucose I	[16]
13	5.41	635	483, 331, 313, 169, 125	Trigalloyl glucose II	[16]
14	5.94	197	182, 167, 153	Syringic acid	[17]
15	6.56	335	320, 291	Digallic methyl ester	[18]
16	6.88	787	635, 483, 331, 169	Tetra galloyl glucose	[19]
17	6.92	301	284, 257, 255, 185	Ellagic acid	[17]
18	7.47	939	787, 635, 169	Penta galloyl glucose	[19]
19	8.22	349	334, 319, 233	Digallic dimethyl ester	[18]
20	8.84	501	486, 471, 457, 349	Trigallic dimethyl ester	[18]

**Table 2:** The susceptibility test of the OGE, Qi-ZnONPs and antibiotics against *H. pylori* ATCC-43526 using the agar diffusion method.

Antimicrobial Tested	Inhibition Zone Diameter (mm)	Potency
OGE	16	Intermediate
Qi-ZnONPs	21	Moderately Suscetible
Amoxicillin	28	Suscetible
Clarithromycin	31	Susceptible

**Table 3:** The minimum inhibitory concentration (MIC) of different combinations of OGE/Qi-ZnONPs and the antibiotics against *H. pylori* (ATCC-43526) using a micro-dilutions method.

Antimicrobial Tested	MIC_90_ Value (µg/mL)	Inhibition %
OGE	37.5	97.8
Qi-ZnONPs	18.75	98.4
Amoxicillin	0.586	93.2
Clarithromycin	0.293	90.7

**Table 4:** Fractional inhibitory concentrations (FIC) of different combinations of the OGE/Qi-ZnONPs and antibiotics against *H. pylori* (ATCC-43526) using the checkerboard micro-dilutions method.

Antimicrobials Combinations	MIC_90_ Value (µg/mL)	FIC Values	Outcome
Amoxicillin: OGE			
1:1	37.50	2.25	Indifference
1:2	37.50	2.25	Indifference
1:4	18.75	1.13	Indifference
1:8	18.75	1.13	Indifference
Amoxicillin: Qi-ZnONPs			
1:1	18.75	1.13	Indifference
1:2	9.38	0.563	Indicated additives
1:4	4.69	0.282	Synergy
1:8	4.69	0.282	Synergy

## Data Availability

The data is contained within the article and Supplementary Materials.

## Supplementary Materials

The following supporting information can be downloaded at: https://www.mdpi.com/article/10.3390/molecules27144559/s1, Figure S1: UV analysis of QI-ZnONPs. Figure S2: Zeta sizer diagram (A) and zeta potential diagram (B) of Qi-ZnONPs.
