# Supplementary material for: Synergistic Effect between Amoxicillin and Zinc Oxide Nanoparticles Reduced by Oak Gall Extract against Helicobacter pylori
Source: Molecules. 2022 Jul 17;27(14):4559. doi: 10.3390/molecules27144559 (PMC9320066; doi:10.3390/molecules27144559)
Supplement: Supplementary file 1 [file molecules-27-04559-s001.zip › molecules-1779802-supplementary.pdf]

Supplementary data

Synergistic effect between amoxicillin and biosynthesized zinc oxide nanoparticles based on *Quercus infectoria* galls against *Helicobacter pylori*

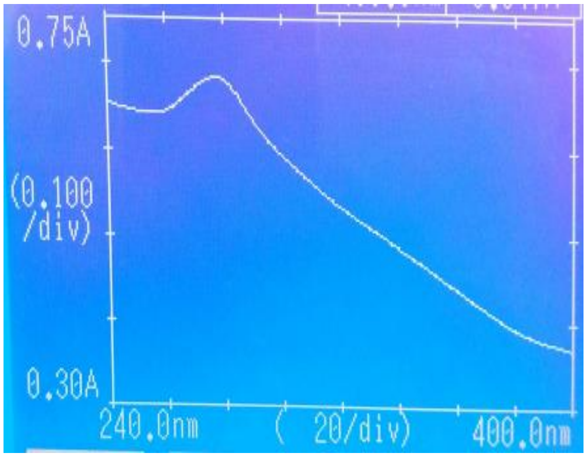

Figure S1. U.V analysis of QI-ZnONPs.

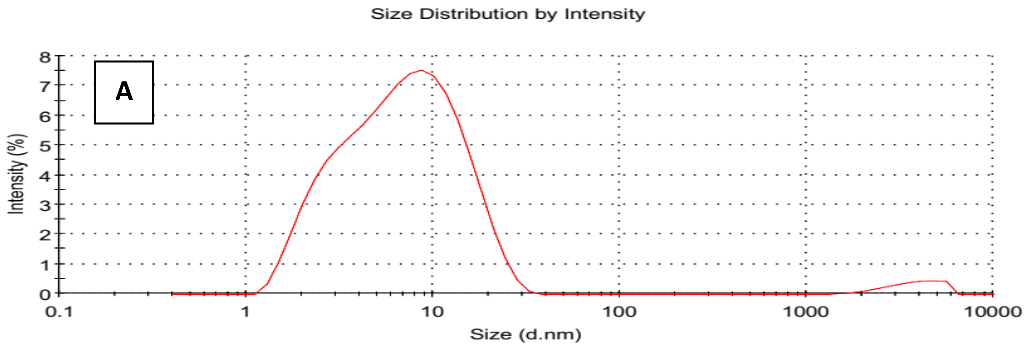

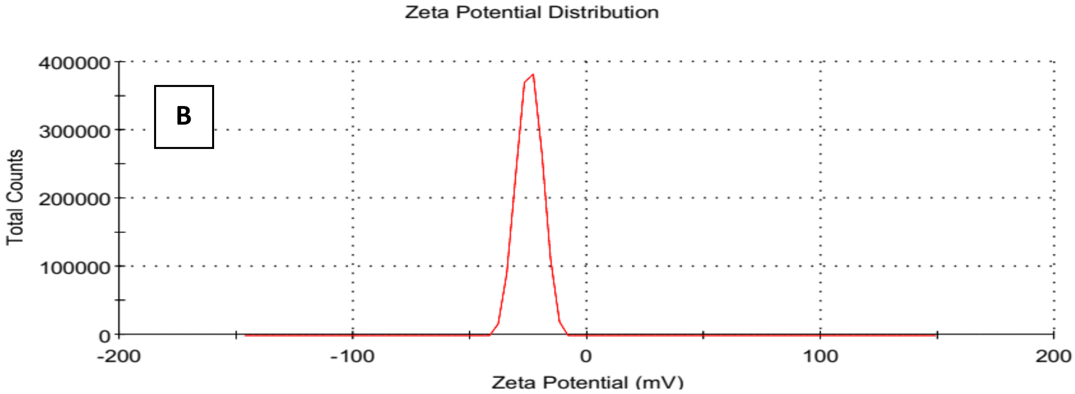

Figure S2. Zeta sizer diagram (A) and zeta potential diagram (B) of Qi-ZnONPs.
